# Measles virus: evidence of an association with Hodgkin's disease

**DOI:** 10.1038/sj.bjc.6601900

**Published:** 2004-06-29

**Authors:** D Benharroch, Y Shemer-Avni, Y-Y Myint, A Levy, E Mejirovsky, I Suprun, Y Shendler, I Prinsloo, S Ariad, B Rager-Zisman, M Sacks, J Gopas

**Affiliations:** 1 Department of Pathology, The Soroka University Medical Center, PO Box 151, Beer-Sheva 84101, Israel; 2 Department of Virology, The Faculty of Health Sciences, Ben-Gurion University of the Negev, PO Box 653, Beer-Sheva 84105, Israel; 3 Department of Epidemiology, the Faculty of Health Sciences, Ben-Gurion University of the Negev, PO Box 653, Beer-Sheva 84105, Israel; 4 Department of Pathology, Nahariya Hospital, PO Box 21, Nahariya 22100, Israel; 5 Department of Oncology, The Soroka University Medical Center, PO Box 151, Beer-Sheva 84101, Israel; 6 Department of Immunology and Microbiology, The Faculty of Health Sciences, Ben-Gurion University of the Negev, PO Box 653, Beer-Sheva 84105, Israel

**Keywords:** Hodgkin's disease, measles virus, Epstein–Barr virus

## Abstract

The quest for an infectious agent that may account for cases of Hodgkin's disease (HD) especially in young adults has proven vain until lately. We have recently reported findings that suggested the presence of measles virus (MV) antigens and MV RNA in the tissues of patients with HD. Support for an association between MV and HD has been provided by recent epidemiological findings relating the occurrence of HD to exposure to measles in pregnancy and the perinatal period. We now present further evidence of this putative association based on immunohistochemical, reverse transcriptase–polymerase chain reaction (RT–PCR) and *in situ* hybridisation studies (ISH) on HD tissues. Biopsies from 82 (54.3%) of our cohort of 154 patients showed a positive immunostain with at least two of the anti-measles antibodies used. Latent membrane protein-1 immunostaining for Epstein–Barr virus was positive in 46 (31.1%) of the patients examined. Reverse transcriptase–PCR and ISH for measles RNA were positive in seven and 10 of 28 patients, respectively. Preliminary clinicopathological associations between MV and HD are noted in this study, but no causal relationship can be claimed at this stage.

An association between Hodgkin's disease (HD) and infection with Epstein–Barr virus (EBV) is now well established on the basis of immunohistochemical (IHC) and *in situ* hybridisation (ISH) studies, and causal implications of this association have been inferred (Knecht *et al*, 2001; Niedobitek *et al*, 2001; Jarrett, 2002). Young adult HD patients from a high socioeconomic environment do not, however, usually have an EBV-associated disease (Alexander *et al*, 2000), although epidemiological evidence suggests that this age group is the one whose HD is most expected to be related to late exposure to a common infectious agent (Gutensohn and Cole, 1981; Glaser, 1990; Westergaard *et al*, 1997; Sleckman *et al*, 1998; Glaser *et al*, 2002). Evidence of delayed exposure to EBV is present in only a minority of these young adult HD patients (Jarrett, 2002). The suggestion that EBV-negative cases of HD had previously been EBV positive (the hit-and-run hypothesis) or that a defective EBV genome is integrated in the chromosomal DNA of EBV-negative tumour cells in HD has not been confirmed (Delecluse *et al*, 1997; Brousset, 2002; Gallagher *et al*, 2003).

Attempts to demonstrate other infectious agents, including CMV, HHV-6, -7 and -8, polyoma JC virus, SV40, lymphotropic papova virus, HTLV-1 and -2 and human retrovirus 5 in HD patients, have failed (reviewed in Torreli *et al*, 1991; Armstrong *et al*, 1998; Berneman *et al*, 1998; Cozen *et al*, 1998; Jarrett and MacKenzie, 1999; Schmidt *et al*, 2000; Gallagher *et al*, 2002).

We have previously suggested that measles virus (MV) may be associated with HD (Benharroch *et al*, 2001; Gopas *et al*, 2001; Benharroch *et al*, 2003). This lymphotropic virus also shows tropism to the respiratory tract, which may explain the frequent involvement of cervical and mediastinal lymph nodes in HD. Following the introduction of vaccination against MV, measles may appear at an older age than expected (Veit *et al*, 1991; Desai *et al*, 2002). This may represent late exposure to a common infectious agent and account for the constant incidence of HD, at least in some countries, in the postmeasles vaccination era. In our original studies, we found an apparent epidemiologic association between exposure to outbreaks of measles in girls aged 10 and 16 years and an increased risk of HD in these patients in young adulthood (Gopas *et al*, 2001). We also described IHC evidence for the presence of MV antigens in 60.3% of HD lymph nodes examined. Our cohort was later expanded, and expression of MV antigens was found in 105 (73.4%) of HD patients. We also demonstrated MV RNA sequences in two of 19 biopsies from our HD patients (Benharroch *et al*, 2003).

In the present study, we report further IHC, reverse transcriptase–polymerase chain reaction (RT–PCR) and ISH evidence for the putative association between MV and HD. We also describe certain preliminary clinical associations with the expression of MV and EBV in the tissues of HD patients.

## MATERIALS AND METHODS

The study was performed on a cohort of 154 patients with classic HD diagnosed in the years 1975–2000 at the Soroka University Medical Center in Beer-Sheva, from whom tissues were available for investigation. This is an expanded cohort of the patients described in our preliminary studies (Benharroch *et al*, 2003). Only primary (pretreatment) biopsies in which the diagnosis and histological type were confirmed by two haematopathologists (DB and MS) were considered. Clinical data and follow-up data were retrieved from the files of the patients or, when these were not available, were obtained from the Israel Population Registry by linkage.

### Immunohistochemistry

Immunohistochemical studies were performed on paraffin-embedded tissues using the avidin–biotin peroxidase complex method with the Vectastain kit of Vector Laboratories (Burlingame, CA, USA), as described previously (Benharroch *et al*, 1995). Latent membrane protein-1 (LMP1) antibody for the EBV antigen from Dako (Glostrup, Denmark) was used. The anti-MV antibodies employed (anti-nucleoprotein (NP)-MV, anti-haemagglutinin (HA)-MV, anti-matrix (M)-MV and anti-phosphoprotein (P)-MV) were purchased from Chemicon International Inc. (Temecula, CA, USA). The last two were studied in isolated cases only. In addition, anti-NP (H14; L39/22; L39/61) and anti-HA (K83; L77) antibodies were made available to one of us (BR) (H14 from Birrer *et al* (1981) and the others from Schneider-Schaulies *et al* (1992)). The specificity of these anti-NP-MV antibodies had been established by Western blotting.

The IHC stains were considered positive if 10% or more of the Hodgkin–Reed–Sternberg (HRS) cells were stained. In order to increase the stringency of the assay, a case was considered positive for MV, if at least two of the NP and/or HA antibodies mentioned above were positive. A murine neuroblastoma cell line permanently infected by MV (Gopas *et al*, 1992) was used as a positive control and the same cell line, but without infection with MV, was used as a negative control. These cell lines were also used as controls for the RT–PCR and ISH studies. In all, 25 sections from cases of non-Hodgkin's lymphomas (NHL) were also examined by IHC.

### RT–PCR analysis

Total RNA was extracted from frozen lymph node samples from a subset of our HD patients, in whom frozen tissue from a primary biopsy was available. For this purpose, we used EZ-RNA Total RNA Isolation kit (Biological Industries Co., Beit Haemek, Israel) according to the manufacturer's instructions. In all, 40 U of RNA-guard (Promega, Madison, WI, USA) were added to each sample. One-step RT–PCR was carried out with Ready To Go™ RT-PCR beads (Amersham Pharmacia Biotech Europe GmbH, Freiburg, Germany). A measure of 3 *μ*l of RNA (2–6 *μ*g) was used for each reaction. The primers used in this study are shown in Table 1

**Table 1:** Primers used for nested RT–PCR

**Gene region amplified**	**Primer symbol**	**Primer sequence**	**Location in region**	**Product size**
HA	HM3 (upstream/outer)	CAGTCAGTAATGATCTCAGC	8106–8125	596
	H6 (downstream/outer)	CTTGAATCTCGGTATCCACTCCAAT	8677–8701	
	H7 (upstream/inner)	GAGCTCAAACTCGCAGCCCTTTGTC	8147–8171	336
	H4A (downstream/inner)	ATCCTTCAATGGTGCCCACTCGGGA	8458–8482	
				
NP	NPM2 (upstream/outer)	GGGATATCCGAGATGGCCACACTTTTAAGG	96–125	529
	NPMRT (downstream/outer)	GGGCTAGGATGGATCCCAGAATCATGTTG	625–597	
	NPM3 (upstream/inner)	GGGTCTTGCACTTCAATATCTGAG	105–127	482
	NPM4 (downstream/inner)	GGGTCTTGCACTTCAATATCTGAG	586–563	
				
pp2C*β*	8.861F	GGG,AAG,TCG,AGA,TAA,CAT,GAG	16 151–16 171	290
	9.576R	CCCATCACTTTCTCTATGTG	25 480–25 460	

RT–PCR=reverse transcriptase–polymerase chain reaction; HA=haemagglutinin; NP=nucleoprotein; pp2C*β=*protein phosphatase 2C*β*.

. Nested PCR was performed using SUPER-NOVA DNA polymerase (Roche Molecular Biochemicals, Mannheim, Germany).

RNA was also extracted from several paraffin-embedded tissue sections from HD patients, using the Paraffin Block RNA Isolation kit (Ambion Inc., The RNA Company, Austin TX, USA) and RT–PCR followed by nested PCR was performed with 10 *μ*l of extracted RNA as described above.

In addition to the neuroblastoma cell lines mentioned above, seven cases of NHL and a case of HD shown to be negative for MV by IHC were included as controls. The quality of the RNA preparations was tested by amplification of transcripts of the housekeeping genes, *β*-actin and protein phosphatase 2C *β* (pp2C*β*).

To confirm our RT–PCR findings, the cDNA products of the nested PCR were blotted to GeneScreen (NEN Research Products, Boston, MA, USA) and hybridised to either an NP-MV probe (CAATCCCTGGAGATTCCTCA) or to an HA-MV probe (GCTGGAAGCTGACACCTTTC), labelled with [*γ*-^32^P]ATP (Amersham).

### ISH on paraffin sections

For the preparation of digoxigenin (DIG)-labelled antisense and sense probes to MV-specific mRNAs, the plasmids containing NP-MV encompassing positions 105–586 and those containing HA-MV encompassing positions 8147–8482 in PGEM-T Easy Vectors (Promega) were linearised with *Sal*I or *Sac*II. *In vitro* transcription was performed with the DIG-RNA Labelling kit, using SP6 for NP antisense and HA sense probes and T7 for NP sense and HA antisense DIG-labelled probes. The quality of the probes was determined by dot blotting using a DIG-DNA detection kit (Roche Molecular Biochemicals) according to the manufacturer's instructions with minor modifications.

*In situ* hybridisation was carried out according to the method of Ogata *et al* (1997) with certain modifications. Paraffin sections were deparaffinised, rehydrated and treated by microwave in the presence of 10 mM MgCl_2_ buffer (pH 6) for 5 min at 750 W. Sections were allowed to cool for 20 min, and then digested with 20 *μ*g ml^−1^ proteinase K for 10 min at 37°C. They were then fixed in 4% paraformaldehyde in 0.1 M phosphate buffer, acetylated, dehydrated and air-dried. Hybridisation was performed overnight at 50°C with DIG-labelled RNA in hybridisation buffer containing 50% formamide, 10 mM Tris-HCl (pH 7.6), 200 *μ*g ml^−1^ tRNA, 1 × Denhardt's solution, 10% dextran sulphate, 600 mM NaCl, 0.25% SDS and 1 mM EDTA, pH 8.0. Hybridised DIG-labelled probes were detected using the DIG-Nucleic Acid Detection kit (Roche Molecular Biochemicals), following blocking with 1.5% of the blocking reagent included in the kit. The colour reaction was stopped with 10 mM Tris-HCl (pH 7.6) and 1 mM EDTA. Sections were then fixed in 4% paraformaldehyde in phosphate-buffered saline and stained with haematoxylin. In addition to the neuroblastoma cell lines used as positive and negative controls, HD cases negative for MV by IHC and NHLs served as controls.

### Comparison between IHC, RT–PCR and ISH

This was performed on a sample of cases. For this comparison, IHC positivity to a single NP-MV or HA-MV antibody was considered sufficient.

### Clinicopathological correlations

A clinicopathological study on MV and EBV expression in HD is under way. Some of the preliminary findings are presented here, but the complete study will be described in a separate publication. For contingency table analysis, the *χ*^2^ or Fisher's exact test was used, as appropriate.

## RESULTS

Biopsies were obtained from 90 male and 64 female patients with untreated classical HD. Their ages ranged from 4 to 86 (mean 35.6±17.2 and median 33.00) years.

The biopsies were classified as nodular sclerosis in 88 (57.1%) patients, mixed cellularity in 57 (37%), lymphocyte-rich classical HD in two (1.3%), lymphocyte depleted in two (1.3%) and undetermined HD in five (3.3%).

### Immunohistochemistry

Cytoplasmic LMP1 immunostaining was positive in 46 (31.1%) of 147 cases. The IHC expression of MV antigens is shown in Table 2

**Table 2:** Immunohistochemical documentation of the expression of various measles virus antigens in tumor cells

	***n***	**Positive *n* (%)**	**Negative *n* (%)**
*Nucleoprotein*			
H14	149	67 (45)	82 (55)
L39/22	130	80 (61.5)	50 (38.5)
L39/61	141	51 (36.2)	90 (63.8)
MNP (Chemicon)	56	30 (53.6)	26 (46.4)
			
*HA*			
K83	81	25 (30.9)	56 (69.1)
L77	84	32 (38.1)	52 (61.9)
H2 (Chemicon)	23	14 (60.9)	9 (39.1)

HA=haemagglutinin.

and Figures 1

**Figure 1:**
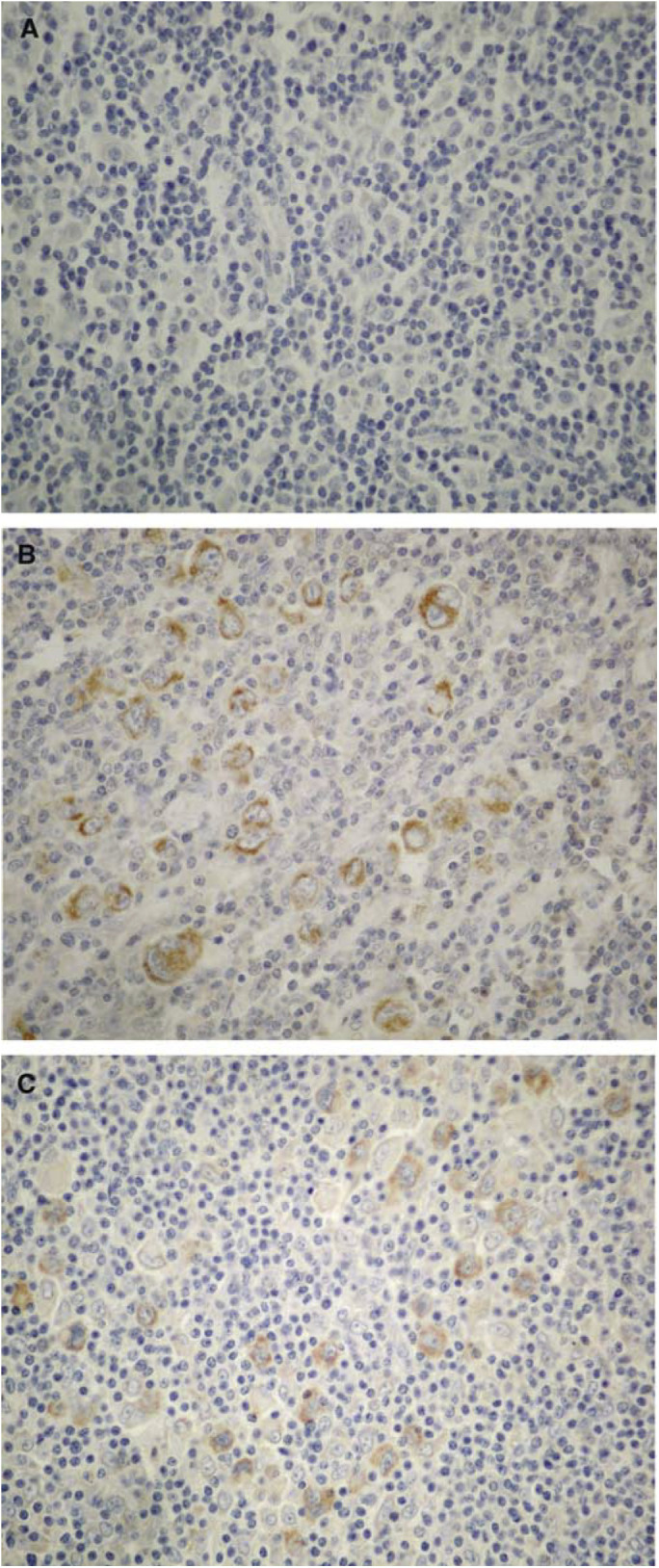
IHC studies for the NP-MV antigens (IHC with diaminobenzidine, × 430): (**A**) HD case, negative with the L39/22 anti-NP antibody. (**B**) HD case positive with the L39/22 anti-NP antibody in HRS cells. (**C**) HD case positive with the L39/61 anti-NP antibody in HRS cells.

and 2

**Figure 2:**
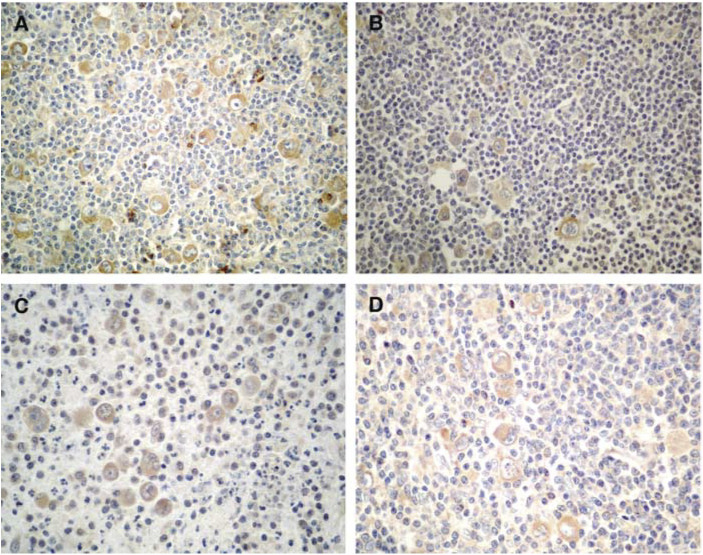
IHC studies for other MV antigens (IHC with diaminobenzidine, × 430): (**A**) HD case positive with the L77 anti-HA antibody in HRS cells. (**B**) HD case positive with the H2 anti-HA antibody in HRS cells. (**C**) HD case positive with an anti-matrix antibody in HRS cells. (**D**) HD case positive with an anti-phosphoprotein antibody in HRS cells.

. According to our definition of positivity, 82 (54.3%) cases were positive and 69 (45.7%) were negative for MV antigens. Immunostaining was cytoplasmic. It was granular in some cases, mainly with the L39/22 anti-NP antibody. Staining was never noted in the cell membrane even with anti-HA antibodies. Fixation in B5 gave better results than neutral formalin. We found that a case that showed positivity for one of the HA antigens had a 90.2% chance of being positive for an NP antigen, but only 34.9% of NP-positive HRS cells were also HA positive. All the NHL cases studied were negative for MV antigens.

### Detection of MV nucleic acids

Reverse transcriptase–polymerase chain reaction and nested PCR were performed on frozen tissues from 16 HD cases and were positive for HA-MV in three cases, positive for both HA-MV and NP-MV in an additional case and negative for both HA-MV and NP-MV in 12 cases. In all the positive cases and in 10 of the negative cases, the housekeeping gene pp2C*β* showed the presence of good quality RNA.

Reverse transcriptase–polymerase chain reaction for HA-MV was also performed on RNA extracted from paraffin sections in 12 additional cases in which no frozen tissue was available. It was positive in three cases and negative in nine. As negative controls, we used a case of HD that was negative for MV by IHC as well as seven cases of various NHL ranging from small lymphocytic lymphoma to diffuse large B-cell lymphoma. All these controls were negative for HA-MV and NP-MV. The majority of the control cases (85%) were positive for pp2C*β*.

#### Southern blot analysis

**Figure 3:**
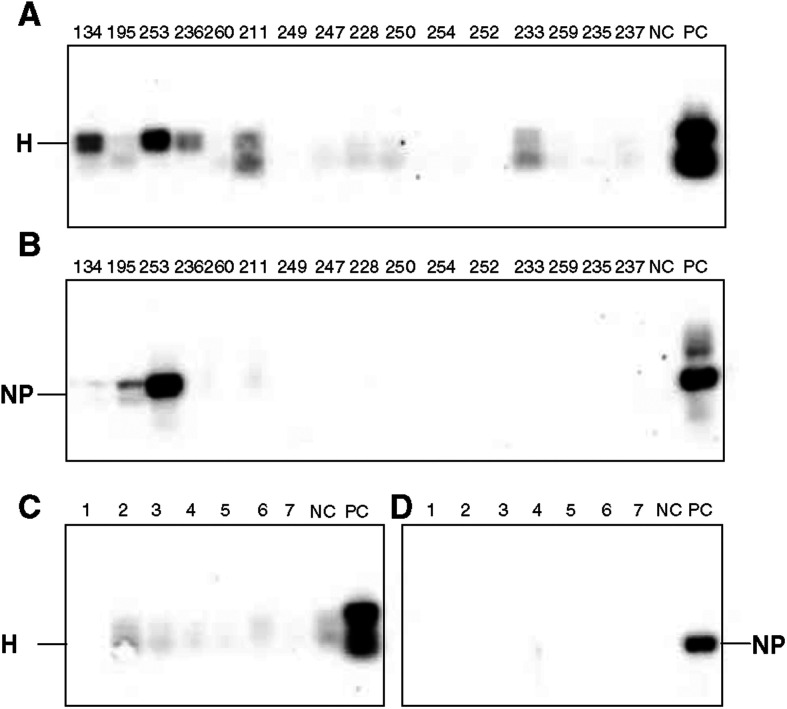
Southern blot using cDNA products of an RT–PCR performed to detect MV-RNAs: (**A**) and (**C**) were hybridised with probes for HA-MV; (**B**) and (**D**) with probes for NP-MV. (**A**) and (**B**) show HD cases (PC-positive control – the neuroblastoma cell line persistently infected with MV; NC-negative control – the same cell line, not infected by MV). (**C**) and (**D**) show NHL cases.

cDNA from the RT–PCR studies were used. HA-MV was positive in four and negative in 11 cases. NP-MV was positive in two and negative in 14 cases. The seven cases of NHL were negative for MV (Figure 3

).

#### Sequencing

**Figure 4:**
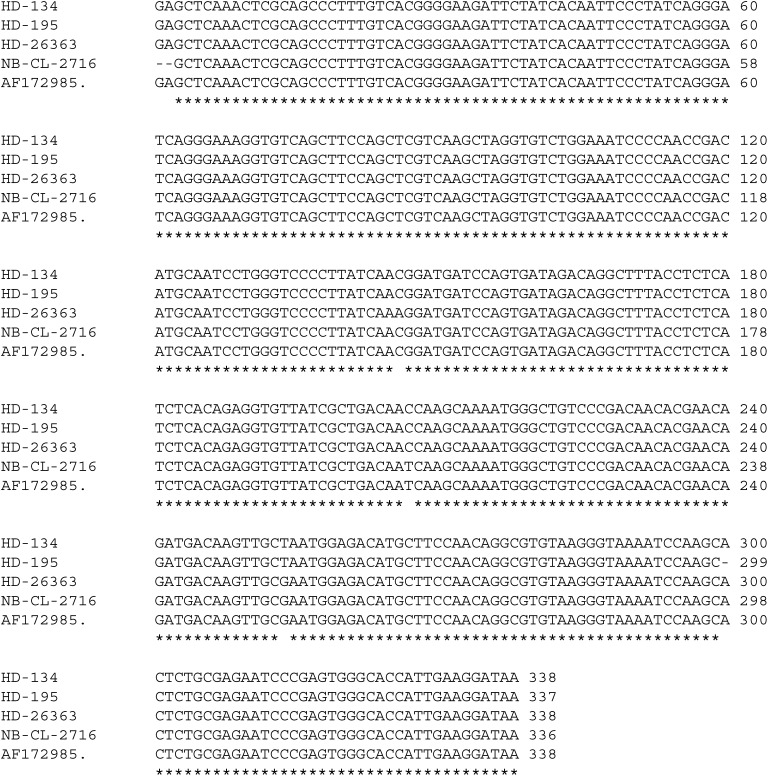
Alignment of MV-HA cDNA sequences isolates from three HD patients. NB-CL-2716 is a neuroblastoma cell line persistently infected by the Edmonston strain of MV. AF172985 is the GenBank accession number of the sequence of the Edmonston strain of the MV. Asterisks indicate single-nucleotide differences in the sequence.

Cloning and sequencing were performed in three cases that were positive for HA-MV and almost complete homology with the control strain of the MV (GenBank accession number – AF172985) was found (Figure 4

).

### *In situ* hybridisation (Figure 5)

**Figure 5:**
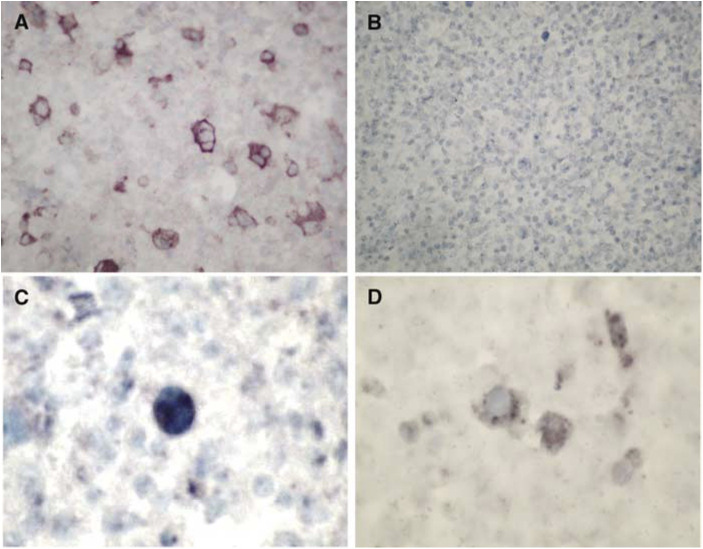
ISH (× 430): (**A**) Positive control, the 2716 neuroblastoma cell line infected by MV. (**B**) HD case negative for MV by IHC. (**C**) HD case positive for HA-MV RNA. (**D**) HD case positive for NP-MV RNA.

NP-MV was positive in five and negative in 13 cases of HD. In the positive cases, the signal was located in the cytoplasm of HRS cells. In three additional cases, both N- and C-terminal NP-MV were positive. HA-MV was positive in two and negative in five cases. The two neuroblastoma cell lines were confirmed as positive and negative controls. Two cases of HD negative for MV by IHC, an uninvolved bone marrow biopsy from an HD patient and the NHL cases studied, were all negative in the ISH studies.

### Comparison between the three assays

The results of the different assays employed are compared in Table 3

**Table 3:** Comparison of results obtained by three assays

	**NP-MV**		**HA-MV**	
	**Positive/*n***	**(%)**	**Positive/*n***	**(%)**
**RT-PCR**	1/16	(6.25)	7/21	(33.3)
**ISH**	7/14	(50)	4/7	(57.1)
**IHC**	25/29	(86.2)	20/29	(68.9)

HA=haemagglutinin; NP=nucleoprotein; MV=measles virus; RT–PCR=reverse transcriptase–polymerase chain reaction; ISH=*in situ* hybridisation studies; IHC=immunohistochemical.

. The rate of positivity was highest for IHC and lowest for RT–PCR in the sample in which the comparison was made.

### Clinicopathological correlations

As indicated above, the HRS cells in 54.3% of our 154 HD patients showed positive immunostaining for MV antigens. Tumour cells were positive for MV in significantly more female than male patients (40 (64.5%) female and 42 (47.2%) male patients – *P*=0.036). Nodular sclerosis HD was more frequently associated with MV than mixed cellularity HD (58 (68.2%) as compared with 21 (40.4%) – *P*=0.0013). All possible combinations of immunostaining for MV and LMP were observed. When patients with positive MV and negative LMP immunostaining (MV+; LMP−) were studied for histologic type and compared with MV−; LMP+ cases, the association of NS-HD with isolated MV positivity was even more striking (*P*=0.00008) (Table 4

**Table 4:** Associations between the expression of MV and EBV (LMP) by histological type of HD

	**Mixed cellularity (%)**	**Nodular sclerosis (%)**
**MV+; LMP−**	11 (19.6)	45 (80.4)
**MV−; LMP+**	14 (66.7)	7 (33.3)
**MV+; LMP+**	10 (47.6)	11 (52.4)
**MV−; LMP−**	15 (42.9)	20 (57.1)

MV=measles virus; EBV=Epstein–Barr virus; LMP=latent membrane protein; HD=Hodgkin's disease.

). The MV+; LMP− combination was also significantly associated with early Ann Arbor stages (*P*=0.023). It is worth noting that more than 35 of the LMP− cases were also MV−.

## DISCUSSION

We have recently suggested that antigens and nucleic acids of the MV may be present in the lymph nodes of HD patients (Benharroch *et al*, 2003). We were aware of methodologic limitations in our initial studies and could not exclude the possibility that the MV may grow preferentially in the large tumour B (HRS) cells of this disease. However, the IHC evidence for the presence of the MV antigens, including several of the MV proteins, was impressive. On the other hand, the yield of the RT–PCR analysis was low. There are several possible explanations for this finding, the most likely being a low MV-RNA content in the HRS cells or the high content of RNAses in the eosinophils that are usually numerous in HD tissues (Benharroch *et al*, 2003).

Considering a causal relationship between the MV and HD on the basis of these findings would have been premature (Benharroch *et al*, 2003), and it should be remembered that it took decades to establish such a link between EBV and HD and some still refute its validity (Harris, 1999). The presence of persistent MV infection in tissues other than the central nervous system is still questioned (Griffin, 2001). It is most probable that only persistent infection would explain a malignant tumour developing years following MV infection. Although MV is not considered an oncogenic virus, several reports have suggested an epidemiological association not only between MV and brain tumours but also with HD (Dickinson *et al*, 2002; Tyari *et al*, 2003).

We have now expanded our IHC study by using both commercial and experimental antibodies to the NP, HA, M and P antigens in a larger series of cases of HD. The specificity of some of the antibodies used in this study was established by Western blotting.

We feel that the results of the present study support our previous findings on the expression of MV antigens in the HRS cells in many cases of HD (Benharroch *et al*, 2003). Moreover, the much more marked coexpression of HA-MV in cases positive for NP-MV antigens than the coexpression of NP-MV in cases positive with HA-MV antigens is consistent with the predominance of NP antigens among the MV proteins (Griffin, 2001). The absence of membrane expression of HA-MV antigens may be due to formalin or B5 fixation. It is worth noting that Griffin (2001) described a reduced expression of membranal HA-MV antigens in persistently MV-infected cells.

The small proportion of positive cases with RT–PCR may be due to the low content of MV-RNA in HD tissues because of the low number of HRS cells in the lesions. We should have overcome this difficulty by the use of nested RT–PCR but the intrinsic lability of RNA, especially in archival material, but also in frozen tissues, which had been stored at −70°C for 6 months to 7 years, represents a serious drawback. The possible effect of ribonucleases in the eosinophils may also be responsible for the low yield of MV-RNA by RT–PCR (Slifman *et al*, 1986; Norrback *et al*, 1998; Hamalainen *et al*, 1999). These limitations probably reflect an underestimate of the MV-RNA content of our biopsies.

We nevertheless detected MV-RNA in seven of 28 of our cases of HD by RT–PCR. The Southern blot analysis showed MV-RNA in only six of the seven cases, but this difference may be technical. Sequencing, carried out in four cases positive for HA-MV including one that was positive for NP- and HA-MV supported the above findings and unequivocally confirmed the identity of the virus. The presence of MV sequences was further established by ISH demonstration of MV-RNA in 10 of 28 cases of HD. We have thus demonstrated the presence of the MV in HD biopsies by several independent techniques.

For the reasons discussed above, we were unable to demonstrate an association between positivity of HD cases for MV antigens by IHC and for MV-RNA by RT–PCR or by ISH. However, such an association would depend on the ability of HRS cells to translate mRNA into protein and on the limitations of the different techniques, including the preservation of the available tissues. Further studies are necessary to determine whether these variations are of biological significance.

The finding of an association between the expression of MV antigens and female gender and NS histological type should clearly be regarded as preliminary and needs confirmation in a larger cohort of HD patients. This finding does not seem consistent with the apparent increase of incidence of the NS subset of HD in female patients, which has been reported in some Western countries in recent years (Liu *et al*, 2000). However, the full impact of measles vaccination on the incidence of HD may only be apparent after the complete eradication of measles. In Scandinavian countries, a significant reduction in the incidence of NS HD in young adult females has, in fact, occurred (Merk *et al*, 1990; Foss-Abrahamsen *et al*, 1997), and may perhaps be related to a more stringent control of measles. On the other hand, in spite of the prevailing measles vaccination policy, late exposure to MV occurs in unvaccinated patients and in young adults with waning immunity (Wood and Brunell, 1995). The reported decline in the frequency of measles vaccination in the United Kingdom (Jansen *et al*, 2003) would be counteractive to a decrease in the incidence of HD. In addition, frequent asymptomatic measles infection has been observed during outbreaks in highly immune individuals (Sonoda *et al*, 2002).

Even though our clinicopathological findings are presented at a very early stage, it seems clear that NS-HD is preferentially associated with the MV+; LMP (EBV)− phenotype. However, in more than 20% of our cases, the two viruses are expressed in consecutive sections in each case. Since immunosuppression of limited duration is well documented in measles, a synergistic interaction of MV with EBV may be relevant in terms of pathogenesis in these 20 cases.

In conclusion, we have documented further evidence for the presence of MV antigens and the MV genome in tissue biopsies from untreated HD patients. Our findings are worth noting in view of the recent reports from the United Kingdom, suggesting an epidemiologic relationship between prenatal and perinatal exposure to MV and the occurrence of brain tumours and HD. Further investigations are needed to confirm our findings and to establish their possible pathogenetic significance.
